# Battle of the eternal rivals: restoring functional p53 and inhibiting Polo-like kinase 1 as cancer therapy

**DOI:** 10.18632/oncotarget.1096

**Published:** 2013-07-13

**Authors:** Frank Louwen, Juping Yuan

**Affiliations:** ^1^ Department of Gynecology and Obstetrics, School of Medicine, J. W. Goethe-University, Frankfurt, Germany

**Keywords:** Oncotargets

## Abstract

Polo-like kinase 1, a pivotal regulator of mitosis and cytokinesis, is highly expressed in a broad spectrum of tumors and its expression correlates often with poor prognosis, suggesting its potential as a therapeutic target. p53, the guardian of the genome, is the most important tumor suppressor. In this review, we address the intertwined relationship of these two key molecules by fighting each other as eternal rivals in many signaling pathways. p53 represses the promoter of Polo-like kinase 1, whereas Polo-like kinase 1 inhibits p53 and its family members p63 and p73 in cancer cells lacking functional p53. Plk1 inhibitors target all rapidly dividing cells irrespective of tumor cells or non-transformed normal but proliferating cells. Upon treatment with Plk1 inhibitors, p53 in tumor cells is activated and induces strong apoptosis, whereas tumor cells with inactive p53 arrest in mitosis with DNA damage. Thus, inactive p53 is not associated with a susceptible cytotoxicity of Polo-like kinase 1 inhibition and could rather foster the induction of polyploidy/aneuploidy in surviving cells. In addition, compared to the mono-treatment, combination of Polo-like kinase 1 inhibition with anti-mitotic or DNA damaging agents boosts more severe mitotic defects, effectually triggers apoptosis and strongly inhibits proliferation of cancer cells with functional p53. In this regard, restoration of p53 in tumor cells with loss or mutation of p53 will reinforce the cytotoxicity of combined Polo-like kinase 1 therapy and provide a proficient strategy for combating relapse and metastasis of cancer.

## INTRODUCTION

### Polo-like kinase 1 and the tumor suppressor p53

Since the discovery of Polo kinase in *Drosophila* in 1988 [1], the Polo-like kinase (Plk) family has been attracting enormous attention, both in academia and in pharmaceutical industry. Five members of the Plk family have been discovered in humans and these serine/threonine kinases have emerged as key players by performing crucial functions in the cell cycle, DNA damage response and neuron biology [2-6]. Plk1 is mainly expressed during the late G2 and M phase, where it regulates various stages of mitosis [2,7]. Plk2 is an immediate early response gene and is expressed in early G1, where it controls the entry into S phase [8]. Plk3 is expressed throughout the cell cycle and involved in cellular response to DNA damage [9]. While Plk4 controls centriole duplication [10-12], Plk5 seems to be linked with neuron biology [6].

Plk1, the most thoroughly characterized member among the mammalian Plks, has multiple important roles in mitosis and cytokinesis, such as centrosome maturation, bipolar spindle formation, kinetochore-microtubule dynamics, activation of the anaphase promoting complex, chromosome segregation and execution of cytokinesis [3,4,13]. In line with this multitude of proposed functions, Plk1 localizes to centrosomes, mitotic spindles, kinetochores, the central spindle and midbody [2,14-16]. The Plk1 activity and its Polo-box binding domain (PBD) are required to mediate its localization to mitotic structures [17-21]. It has been recently reported that while dynactin targets Plk1 to kinetochores [22], the cullin 3 (CUL3)-based E3 ubiquitin ligase containing the adaptor KLHL22 ubiquitylates Lys 492 within the PBD and leads to Plk1 dissociation from kinetochore phosphoreceptors [23]. In the absence of KLHL22, Plk1 accumulates on kinetochores, resulting in activation of the spindle assembly checkpoint (SAC) [23].

Plk1 strongly promotes progression of the cell cycle and is responsible for aggressive proliferation of tumor cells, regarded as a cellular proliferation marker [24]. Overexpression of Plk1 enables cells to override checkpoints, leading to genomic instability and promoting cell transformation [7,25,26]. In support of these interesting data, Plk1 is highly expressed in a broad spectrum of human tumors and its expression often correlates with poor prognosis of tumor patients, suggesting its involvement in oncogenesis and its potential as a therapeutic target [3,26]. Interestingly, genome-wide RNAi screening has identified Plk1 as the only kinase selectively required for the viability of activated Ras cancer cells [27]. Moreover, tumor-initiating cells are responsible for tumor maintenance and relapse. Recently, multiple studies have reported that Plk1 is a potential therapeutic target for eliminating tumor-initiating cells in various tumor types [28-32], implying that inhibiting Plk1 could be useful for combating relapse and metastasis of tumors.

Plk1 offers two functional important target domains: a kinase domain at the N-terminus that is closely related to several members of the superfamily of protein kinases, and the unique specific PBD at the C-terminus. Over the years, efforts have been made to identify Plk1 inhibitors, yielding numerous potent compounds that competitively inhibit the catalytic activity and regulatory function of Plk1 [7,33-35]. In concordance with this, several small-molecule inhibitors of Plk1 are currently under clinical trials [7,36-42]. Based on a fluorescence polarization assay, we have identified the natural product thymoquinone (TQ) and its synthetic derivative Poloxin as the first small molecule inhibitors targeting the PBD of Plk1 [43,44]. Poloxin exhibits a high specificity toward the PBD of Plk1, interferes with the intracellular localization of Plk1, induces mitotic arrest and chromosome congression defects [43]. It suppresses proliferation and triggers apoptosis in cancer cell lines and inhibits tumor growth in xenograft mouse models as well [44].

The key tumor suppressor p53, discovered in 1979 [45,46], has become a milestone in cancer biology [47]. p53 has been the focus since the late 1980s, when it became evident that TP53, the gene encoding the p53 protein, was mutated or altered in various human cancers [48,49]. As the guardian of the genome [50], p53 plays crucial roles in DNA repair, cell cycle arrest, apoptosis, senescence, differentiation, cell adhesion, cell mobility, aging, autophagy, cellular metabolism and somatic cell reprogramming of stem-cell biology [51-59]. p53 functions as a tetramer and its N-terminal region consists of an intrinsically disordered transactivation domain and a proline-rich region, followed by the central, folded DNA-binding core domain for sequence-specific DNA binding, a flexible linker, a short tetramerization domain regulating the oligomerization, and finally the regulatory domain at its C-terminus binding DNA nonspecifically [60]. At homeostasis, the steady-state level of p53 is kept low and p53 function is repressed mainly by the negative regulators mouse double minute 2 (MDM2, human ortholog HDM2) and MDMX (human ortholog HDMX) [61]. p53 is activated in response to oncogenic activation, DNA damage, telomere erosion, ribosomal stress, loss of cell-cell or cell-matrix adhesion, hypoxia or other cellular stress [62]. Its activity and cellular level are tightly controlled by a multitude of regulatory proteins, involving diverse posttranslational modifications [63,64]. Loss of p53 function occurs in most human tumors by either mutation of TP53 itself or by inactivation of the p53 signal transduction pathway [52,65,66]. Therefore, awakening the guardian of the genome by drugging the p53 pathway could have wide applications in fighting cancer [66,67].

During our studies on Plk1 we have very often observed that Plk1 crosses the signaling pathways of p53 and vice versa. In the present review we have focused on the intertwined relationship between the key tumor suppressor p53 and the key mitotic kinase Plk1. We have summarized the function of p53 in mitosis under cellular stress. Finally, we have dealt with the impact of p53 on efficacy of Plk1 inhibitors in tumor cells.

### p53 represses the Plk1 promoter, directly and indirectly

In an early report based on the deletion analysis of the Plk1 promoter, several regions have been identified to contribute to the transcriptional regulation of Plk1 [68]. The potential binding sites for transcription factors E2A, AP1, AP2, SP1, NF-Y and NFκB could be identified in a computer-based search [68]. A stretch of 300 base pairs immediately 5' of the transcription start site of the Plk1 promoter contains a CCAAT motif essential for promoter activity [68]. The mRNA expression of Plk1 is low at the G1/S boundary, increases in the S phase, and is maximally expressed during the G2/M transition. Based on promoter luciferase assays, three activating regions have been identified between 35 and 93 base pairs upstream of the transcription initiation site [69]. A repressor element, termed the cell cycle-dependent element and the cell cycle genes homology region (CDE/CHR), is located in the region of the transcription start site in the Plk1 promoter and mutations within this element diminished cell cycle regulation of transcription [69].

**Figure 1:**
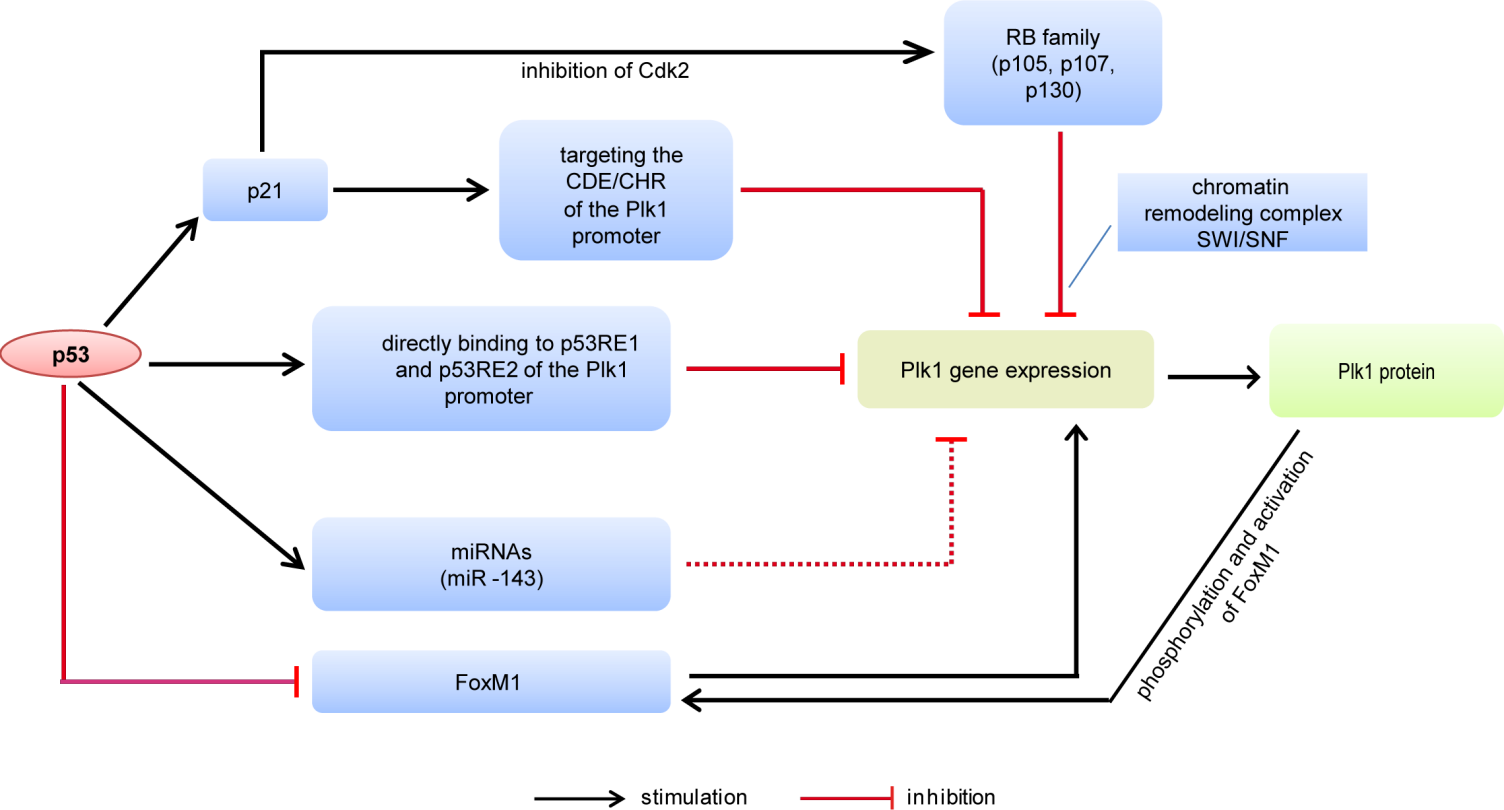
Schematic representation of the Plk1 gene expression controlled by p53 CDE/CHR, cell cycle-dependent element and cell cycle genes homology region. RB, retinoblastoma. FoxM1, forkhead box M1. p53RE, p53 response element. miRNA, micro-RNA.

The Plk1 gene is mainly suppressed by p53 and the retinoblastoma (RB) pathway (Fig. 1). p21/waf1 (p21), the downstream effector of p53, inhibits the Plk1 expression partly by targeting sequences CDE and CHR in the Plk1 promoter [70]. p53 negatively regulates the expression of the forkhead box M1 (FoxM1) [71,72], an oncogenic transcription factor, which stimulates Plk1 expression [73]. On the other hand, the transcriptional activation of FoxM1 is dependent upon the phosphorylation by Plk1 [74]. In addition, the RB family members, p130, p107 and p105, play key roles in transcriptional repression of the Plk1 gene [75,76]. The RB pathway activation results in repression of the Plk1 promoter activity, which is dependent on the chromatin remodeling complex SWI/SNF [75].

Not only indirectly but also directly, p53 regulates the Plk1 expression. Recently, it has been reported that p53 is both necessary and sufficient to mediate a transcriptional repression of the Plk1 promoter [77]. Repression of the Plk1 gene by p53 occurs independently of p21 and of CDE/CHR element upon DNA damage. It is further demonstrated that p53 binds to the Plk1 promoter at two distinct sites termed p53 response element 1 (p53RE1) and p53RE2 based on chromatin immunoprecipitation analysis [77]. Recruitment of p53 to p53RE2, but not to p53RE1, is stimulated in response to DNA damage and/or p53 activation. In addition, wild type p53 represses the promoter expression of Plk1 when fused upstream of a reporter gene [77]. These data strongly suggest that p53 is a major regulator for a proper expression of the Plk1 gene in normal cell cycle progression and upon cellular stresses by controlling the promoter of Plk1, directly or indirectly.

Besides direct transcriptional regulation in the promoter, p53 controls also the target gene expression post-transcriptionally via inducing micro-RNAs (miRNAs), such as miR-34 family [78] and miR-200 family [79]. Recent studies have shown that miR-143, miR-100, miR-593* and miR-10b* target the Plk1 expression in cancer cells [80-85]. It will be of importance to further decipher whether p53 is behind those miRNAs affecting Plk1 expression.

In accordance with the findings from molecular research, it has been reported that immunohistochemical staining of Plk1 in primary breast tumors was significantly associated with the presence of non-functional mutated p53, which predicted a significantly worse survival than those with either Plk1 expression or TP53 mutation alone [86]. More studies are needed to explore if overexpression of Plk1 correlates with loss of p53, non-functional p53 or gain-of-function (GOF) mutant p53 in tumor tissues, and if this correlation is linked to therapy resistance and poor prognosis of tumor patients. In particular, the relationship between the Plk1 expression and GOF mutant p53 should be delineated, since GOF p53 mutants, supported very often by other molecules like Pin1 [87], have widespread genomic locations and profoundly affect gene expression by being tethered by other transcriptional factors to their locations and by binding with p63 to its consensus elements [88,89].

### Plk1 inhibits the function of p53, directly and indirectly

On the other hand, Plk1 is not willing to be obedient with the supervision of p53. Mounting evidence suggests that Plk1 negatively regulates p53 through direct and indirect mechanisms. Plk1 physically binds to the tumor suppressor p53 and inhibits its transactivation activity as well as its pro-apoptotic function in H1299 cells [90]. Immunoprecipitation analyses using a series of deletion mutants of p53 reveal that a sequence-specific DNA-binding region of p53 is required and sufficient for the physical interaction with Plk1. Expression of exogenous Plk1 and p53 in lung carcinoma H1299 cells deficient in p53 greatly decreased the p53-mediated transcription of the p53-responsive p21, MDM2, and BAX promoters, whereas the kinase-deficient mutant Plk1 failed to reduce the transcriptional activity of p53 [90], suggesting that Plk1-mediated negative regulation of p53 might be a fundamental mechanism for the role of Plk1 in oncogenesis. As various point mutations occur most often in the DNA-binding region of p53 in primary cancers, it will be important to define whether and which point mutation in the DNA binding domain of p53 interferes with the interaction of Plk1. It is tempting to assume that mutated p53 is capable of escaping the inhibition of Plk1via interrupted interaction.

**Figure 2:**
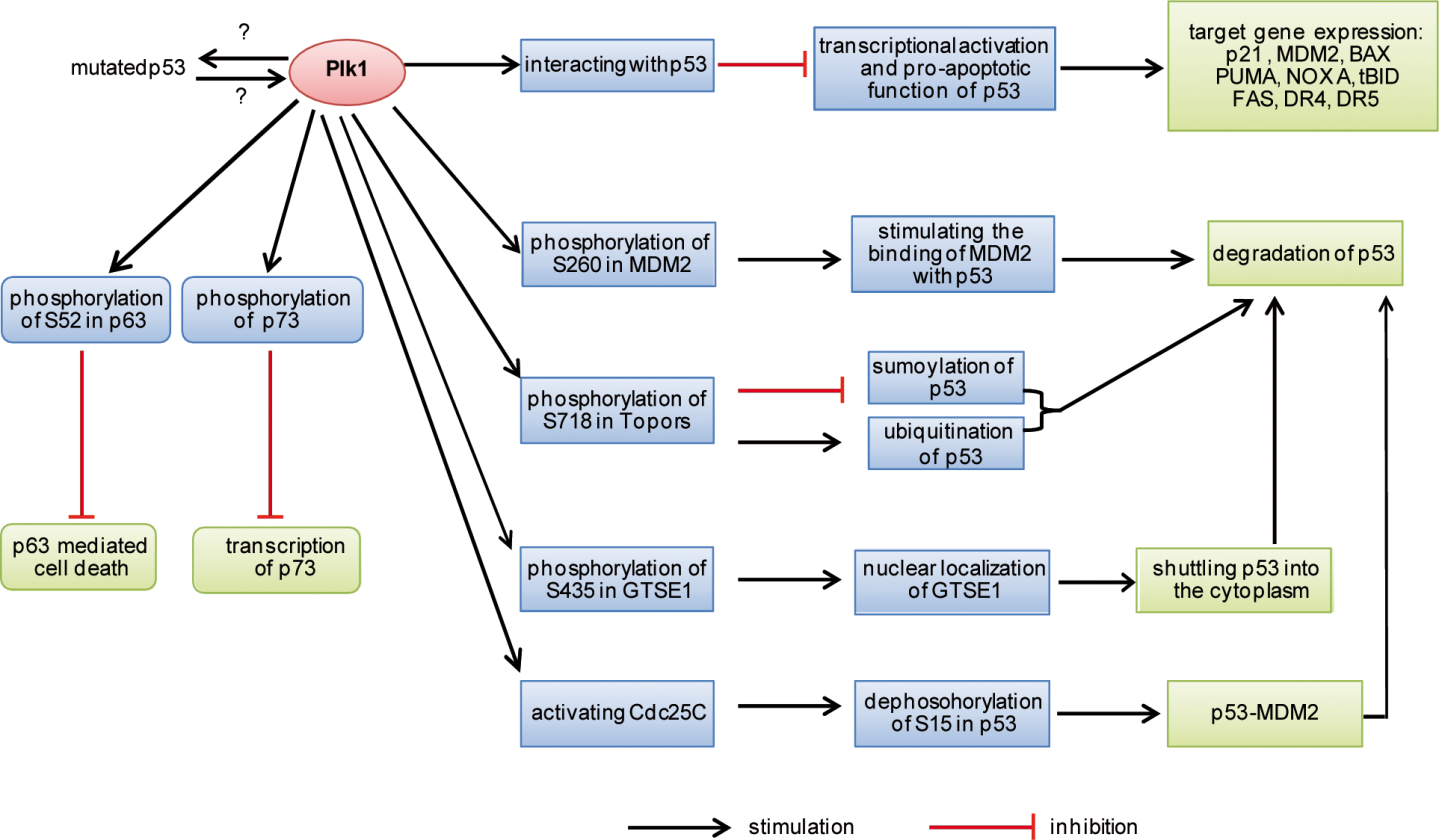
Summary of p53 inactivation by Plk1 MDM2, mouse double minute 2; Topors, topoisomerase I-binding protein. GTSE1, a G2 and S-phase-expressed 1 protein.

p53 inactivation by Plk1 is further underscored by other studies [91-94]. MDM2 is a pivotal E3 ubiquitin ligase and suppresses p53 by proteasomal degradation and transcriptional inactivation [61]. Plk1 phosphorylates S260 in MDM2 and stimulates MDM2-mediated turnover of p53 [93]. Moreover, phosphorylation of S15 in p53 is required for blocking its interaction with MDM2 and contributes to its stabilization [61]. Overexpression of Plk1 decreases phosphorylation of p53 at S15 in UV induced mitotic HEK293 cells via Cdc25C, a phosphatase activated by Plk1 dependent phosphorylation [91]. Thus, this scenario results in inactivation of p53 by Plk1. Furthermore, Plk1 phosphorylates S718 of the topoisomerase I-binding protein (Topors) [92]. Topors has both ubiquitin and SUMO-1 E3 ligase activity and binds also to p53. Expression of a Plk1-unphosphorylatable Topors mutant (S718A) leads to a dramatic accumulation of p53 through blocking its degradation. Plk1-mediated phosphorylation of Topors suppresses the sumoylation of p53, whereas p53 ubiquitination is enhanced, leading to its degradation [92]. In addition, GTSE1, a G2 and S-phase-expressed 1 protein and a negative regulator of p53, is required for the G2 checkpoint recovery. Plk1 phosphorylation of GTSE1 at S435 promotes its nuclear localization and thus shuttles p53 out of the nucleus for its degradation during the recovery [94]. These results are consistent with a previous report that p53 is stabilized in Plk1-depleted HeLa cells [95]. Thus, these data strongly suggest that Plk1 inhibits its rival p53 through either directly physical binding to block its function or indirectly inactivating p53 by promoting its turnover, as illustrated in Fig. 2.

### Plk1 inhibits p73 and p63 in cancer cells lacking functional p53

The battles take place not only between Plk1 and the founding member p53 but also between Plk1 and p73/p63, other two transcription factors of the p53 family. p73 and p63 are expressed as two predominant isoform classes resulting from alternative promoter usage: the TAp73/TAp63 isoforms contain an N-terminal transactivation domain and most resemble p53, while the ΔNp73/ΔNp63 isoforms exhibit a truncated N-terminus [96,97]. It is recently reported that the p73/p63 homeostasis is controlled by a microRNA-dependent circuit [96].

p73 has been implicated in cell cycle regulation, apoptosis and developmental processes [98]. In cisplatin mediated apoptosis in COS7 cells, in which the endogenous p53 is inactivated by SV40 large T antigen, p73 is accumulated in association with a significant down-regulation of Plk1 [99]. Reciprocally, Plk1 reduces the stability of the endogenous p73 and depletion of Plk1 stabilizes p73. p73 is phosphorylated at T27 by Plk1 in kinase assay in vitro [99]. Further analyses demonstrate that p73 binds to the kinase domain of Plk1 through its N-terminal region and wild type Plk1 is able to block the p73-mediated transcriptional activation [99]. Interestingly, Plk1 K82M, a kinase-deficient mutant, binds still to p73 but fails to inactivate the p73-mediated transcriptional activation, suggesting that the catalytic activity of Plk1 is not necessary for the binding but required for the functional inhibition of p73 [99]. Plk1 inhibits p73-mediated transcriptional activity is further supported by another study [100]. In addition, Plk1 knockdown enhances cisplatin chemosensitivity via upregulation of p73 in p53 mutant human epidermoid squamous carcinoma cells [101]. We have also observed that the long-term suppression of Plk1 increases the level of the cyclin dependent kinase inhibitor p21, which is partially induced by elevated tumor suppressor p73 in HeLa cells [102], in which p53 is inactivated via the E6 protein encoded by the oncogenic human papillomavirus (HPV) [103]. Collectively, these findings indicate that Plk1 inhibits p73 by blocking its function and increasing its degradation in cancer cells lacking functional p53.

p63, the remaining member of the p53 family, is unable to get away from the ruling of Plk1. Immunoprecipitation and in vitro pull-down assay reveal that p63 binds to the kinase domain of Plk1 through its DNA-binding region [104]. Plk1 phosphorylates p63 at S52 of the transactivating domain, which is associated with decreased stability of p63 protein and suppressed p63 mediated cell death [104]. Furthermore, Plk1 knockdown in p53-mutated liver tumor cells transactivates PUMA, p21 and 14-3-3sigma, and induces apoptosis [104]. Therefore, Plk1 also controls p63 by phosphorylation and regulates apoptotic cell death in liver tumor cells, in which p53 is inactive.

### Plk1 inhibitors impact both tumor and normal but proliferating cells

Plk1 has been widely established as one of the most promising targets for molecular intervention. Multiple small molecule inhibitors targeting the ATP-binding pocket of the kinase domain and its inactive conformation have been developed [7,33-35]. Among them, BI 2536 and BI 6727 are the most intensively investigated Plk1 inhibitors [105-107]. The results from cancer cell lines and from mouse xenograft models suggest their potent anti-cancer activity [105,107]. In parallel to the kinase domain, the PBD, the regulatory domain of Plk1, has been suggested as a more ideal target due to its unique nature which facilitates the development of specific agents [17]. Indeed, small molecules or peptides targeting the PBD domain of Plk1 have been developed and investigated [43,44,108,109]. The data from Plk1 inhibitors, targeting either the kinase domain or the PBD, are inspiring, based on cell culture systems or xenograft mouse models. However, the clinical results are rather less encouraging by showing limited anti-cancer activity [38,40,110,111]. To identify the molecules and signaling pathways, which are responsible for the cytotoxicity of Plk1 inhibitors, is fundamental for selecting suitable tumor patients for treatment.

It has been widely proposed that Plk1 depletion/inhibition preferentially kills cancer cells compared with normal cells [112-115]. This leads to the hypothesis that Plk1 inhibition is specific and selective by targeting only cancer cells but not normal cells. However, during characterization of the PBD inhibitor Poloxin, we clearly observed that Poloxin inhibited proliferation of a panel of tumor cells as well as several primary/normal non-transformed but proliferating cells, with a comparable efficiency [44]. Proliferation of exponentially normal growing cells, including retinal primary epithelial cells hTERT-RPE1, human umbilical vein endothelial cells (HUVEC), mammary epithelial MTSV-1 cells and fibroblasts, was suppressed upon Poloxin treatment with comparable EC50 values, suggestive of a similar sensitivity of normal cycling cells to Plk1 inhibition. Our observation is supported by the data from other studies of Plk1 inhibitors, such as BI 2536 and the PBD inhibitor purpurogallin (PPG), impacting both cancer cells as well as normal cells with a comparable sensitivity [107,109,116]. Recently, it has been shown that depletion of Plk1 suppresses the viability of MCF10A, a non-transformed mammary epithelial cell line, even more strongly than that of cancer cell line MDA-MB-468 in monolayer cell culture system [117]. Under 3D culture condition, however, MCF10A cells recapitulate epithelial morphogenesis by forming acinar structures and then stop to grow, whereas cancer MDA-MB-468 cells exhibit disorganized structures and continue to proliferate. BI 2536, added to these structures once formed, was effective on MDA-MB-468, and had no effect on MCF10A cells [117]. This is ascribed to the fact that MCF10A cells do not proliferate once the acinar structure is formed. Thus, the sensitivity to Plk1 inhibitors possibly depends on the doubling time of individual cell lines in a monolayer cell culture system and in 3D culture model as well. Given the essential role of Plk1 during mitosis, it is conceivable to propose that Plk1 inhibitors target all rapidly dividing cells irrespective of tumor or normal cells, which is consistent with observed adverse effects of Plk1 inhibitors in clinical trials [38,111,118].

### p53 is pivotal for faithful mitotic progression

Furthermore, it has been reported that Plk1 depletion/inhibition preferentially kills p53 defective cancer cells compared with p53 wild type cancer cells [119,120]. This association of non-functional p53 with sensitivity of Plk1 inhibition leads to the second assumption that inactive p53 facilitates the cytotoxicity of Plk1 inhibition and tumor patients with p53 deficiency/mutation may preferentially benefit from treatment with Plk1 inhibitors.

p53 is localized at centrosomes, mitotic spindles, the centromeres, the midzone/cleavage furrow in mitosis [121-123] and is activated in response to various mitotic stresses such as aberrant spindle formation, abnormal centrosome separation and chromosome damage or missegregation [124-126], suggestive of p53 role in mitosis. p53 knockdown leads to high percentages of cells with abnormal amplification of centrosomes [10,127] and p53 is an important negative regulator of the mitotic kinase Aurora A [128]. p53 localization at the centrosomes in mitosis is ataxia-telangiectasia mutated (ATM)-dependent and monitors mitotic spindle integrity in mitosis [122,129,130], leading to the proposal that ATM and p53 might contribute to the “centrosomal checkpoint”, a network that integrates cell cycle arrest and repair signals [131,132]. p53 is involved in facilitating chromosome segregation to ensure the maintenance of diploid cells [133] and is required for cell cycle arrest after erroneous tetraploid mitosis [134]. Phosphorylation of serine 10 in histone H3 by Aurora B kinase is critical for maintaining normal ploidy, which is coupled with histone deacetylases I/II activity at lysine K9 [135]. Acetylation of K9 and phosphorylation of S10 are interestingly associated in a p53-dependent manner [136]. In addition, p53 is required to enable cells to recover from a nocodazole-induced prometaphase arrest and to coordinate mitotic events [135,136]. The data indicate that p53 is involved in remodeling and reorganizing chromatin structure in mitosis upon stress. Moreover, the spindle assembly checkpoint (SAC) is essential for proper sister chromatid segregation in mitosis. BubR1, an important kinase of the SAC, interacts with and phosphorylates p53 in mitotic cells and regulates protein stability of p53 in mitosis [137]. Mps1, another spindle checkpoint kinase, phosphorylates p53 at T18, which stabilizes and activates p53 in mitosis [138]. This phosphorylation disrupts the interaction with MDM2 and abrogates MDM2-mediated p53 ubiquitination [138]. Mps1 and BubR1 mediated p53 phosphorylation are required for p53 activation to properly induce cell death in a p53-dependent manner in response to mitotic spindle damage [137,138]. Inhibition of Mps1 or BubR1 appears to be disabling a p53-mediated cell death signaling pathway, contributing to accumulation of polyploidy/aneuploidy cells in response to mitotic spindle damage or oncogene-induced DNA damage [137,139]. These data indicate that activation of p53 is essential for protecting cells from genome instability caused by various mitotic failures.

### Inactive p53 is not a predictor for the efficacy of Plk1 inhibitors

We were wondering if the p53 status is indeed a key determinant for the cytotoxic response to Plk1 inhibition in cancer cells. To address this issue, we have examined the cytotoxicity of Plk1 inhibitors/depletion in various cancer cell lines with or without functional p53 [126]. We observed that cancer cells without p53 displayed no increased cytotoxicity upon treatment with different Plk1 inhibitors or siRNA against Plk1. In fact, cancer cells with wild type p53 showed more apoptosis upon Plk1 inhibition, compared to cancer cells without p53 [126]. Our observation is in line with the study by Sur and colleagues that cancer cell lines with or without p53 displayed only minor difference in the sensitivity of the Plk1 inhibitor BI 2536 [140], arguing against a direct role of defective p53 in the response to Plk1 inhibition. Moreover, we examined whether mitotic stress impacted the efficacy of Plk1 inhibitors in cancer cells with or without p53. In the presence of mitotic stress induced by different agents, HCT116 p53+/+ cells displayed a strong apoptosis after treatment with Plk1 inhibitors with increased pro-apoptotic protein Bax, whereas HCT116 p53−/− cells arrested in mitosis with activation of the spindle checkpoint and DNA damage, followed by a mild apoptosis with enhanced anti-apoptotic proteins Bcl-2 and Mcl-1 [141]. Moreover, the surviving HCT116 p53−/− cells showed a strong capability of colony formation. Thus, under severe mitotic stress induced by combined therapy, HCT116 p53+/+ cells conduct apoptosis in mitosis or exit mitosis into the G1 tetraploidy followed by p53-dependent apoptosis, whereas HCT116 p53−/− cells arrest in mitosis, possibly to initiate another round of DNA replication as suggested [142,143]. In this regard, Plk1 inhibition in cancer cells with inactive p53 could lead to an accumulation of polyploidy/aneuploidy, due to the lack of p53-mediated cell death signaling pathway. Taken together, we suggest that loss of p53 is not directly associated with the sensitive cytotoxicity of Plk1 inhibition. Further investigations are required to study whether the long-term outcomes of losing p53, such as compromised or defective DNA damage checkpoint, abnormal metabolism and low differential grade, which possibly make the survival of tumor cells more dependent on Plk1 function, are responsible for the cytotoxicity of Plk1 inhibition.

### Combination of inhibiting Plk1 and restoring p53 as cancer therapy

Whereas inhibiting the initial phases of the cell cycle is likely to generate viable quiescent cells, targeting mitosis offers possibility for killing cancer cells [144]. Mitosis is the most vulnerable phase of the cell cycle, during which it is susceptible to induce cell death with various insults. Microtubule poisons have been proven to be efficacy in clinic against a broad range of malignancies, yet they affect both dividing and non-dividing cells inducing unwished side-effects [145]. It is therefore much desired to develop a new generation of anti-mitotic drugs which target key proteins with specific functions in mitosis, such as Plk1 [7,144] or mitotic centromere-associated kinesin [146]. Several clinical trials of the Plk1 inhibitor BI 2536 have been performed in different tumor types [38,39,110,118] and the mono-therapy of Plk1 inhibitors has shown modest efficacy [38,39,111], suggestive of combined strategy. Interestingly, retinoids enhance the effectiveness of Plk1 inhibitor GSK461364 [147]. In xenograft mouse models, administration of BI 2536 combined with doxorubicin and cyclophosphamide leads to a faster complete response compared with chemotherapy alone and prevents relapse in the poor prognosis-associated triple-negative breast cancer [117]. Plk1 inhibition suppresses proliferation and enhances radiation sensitivity in medulloblastoma cell lines [30]. These data are in consistence with our data that the combination of Plk1 inhibition with anti-mitotic or DNA damaging agents triggers more apoptosis and inhibits more strongly proliferation of cancer cells compared to the mono-treatment [102,141,148,149]. In particular, both Plk1 inhibitors and microtubule-inhibitory agents prolong mitotic arrest, promoting p53 stabilization, Bax expression, caspase activation and apoptosis induction [126,150-152]. In fact, the first clinical trial of Plk1 inhibitor BI 2536 combined with DNA damage agent pemetrexed demonstrates an encouraging antitumor activity in relapsed non-small-cell lung cancer [153]. The combination will synergistically generate cytotoxicity and reduce unwished side-effects of both Plk1 inhibitor and anti-mitotic or DNA damage agent by reducing the dosage of each drug.

The frequent inactivation of p53 in tumors fosters the attractive notion that its functional restoration would constitute an effective tumor-specific therapy. Strategies aimed at restoring wild type p53 function in tumors with p53 loss, mutation or inhibition have been actively pursued and some have already reached clinical trials [67]. The therapeutic impact of those strategies in human cancer has been recently modeled in mice where a clear, even if limited, therapeutic benefit of p53-targeted therapies is established. Thus, restoration of p53 is a powerful strategy for molecular cancer therapy. In tumor cells with wild type p53 or wild type but inactive p53, such as HeLa cells, Plk1 inhibition induces mitotic cellular stress and activates p53 leading further to a strong induction of apoptosis [126]. In tumor cells with loss or mutation of p53, restoration of p53 will empower the cytotoxicity of Plk1 inhibitors by strongly inducing apoptosis. Furthermore, it is conceivable to suggest that restoration of p53 and inhibition of Plk1 will synergistically hinder relapse and metastasis of cancer, as p53 controls stem cell reprogramming and Plk1 inhibition eliminates tumor-initiating cells [28-31]. Finally, it has been reported that BI 2536 generates aneuploidy in primary cardiac fibroblasts [116]. Thus, reactivation of functional p53 will be able to prevent the genome instability caused by Plk1 inhibitors in surviving tumor or normal but proliferating cells. Collectively, reinstallation of functional p53 in tumor cells with loss or mutation p53 will brighten the way for a high efficiency of Plk1 inhibition, combined with anti-mitotic or DNA damage agents. This strategy will fight cancer with a more powerful efficacy by targeting not only proliferation but also relapse and metastasis of tumor cells. Since normal cells have wild type p53, more investigations are needed to identify predictive biomarkers for each combination to maximize efficacy and minimize side-effects in the context of administration schedules, such as succession and dosage, in individual tumors based on the molecular working mechanisms. Hopefully, we will reach the goal of cancer therapy that “the wolves are sated, and the sheep are intact” [154,155].

## SUMMARY

**Figure 3:**
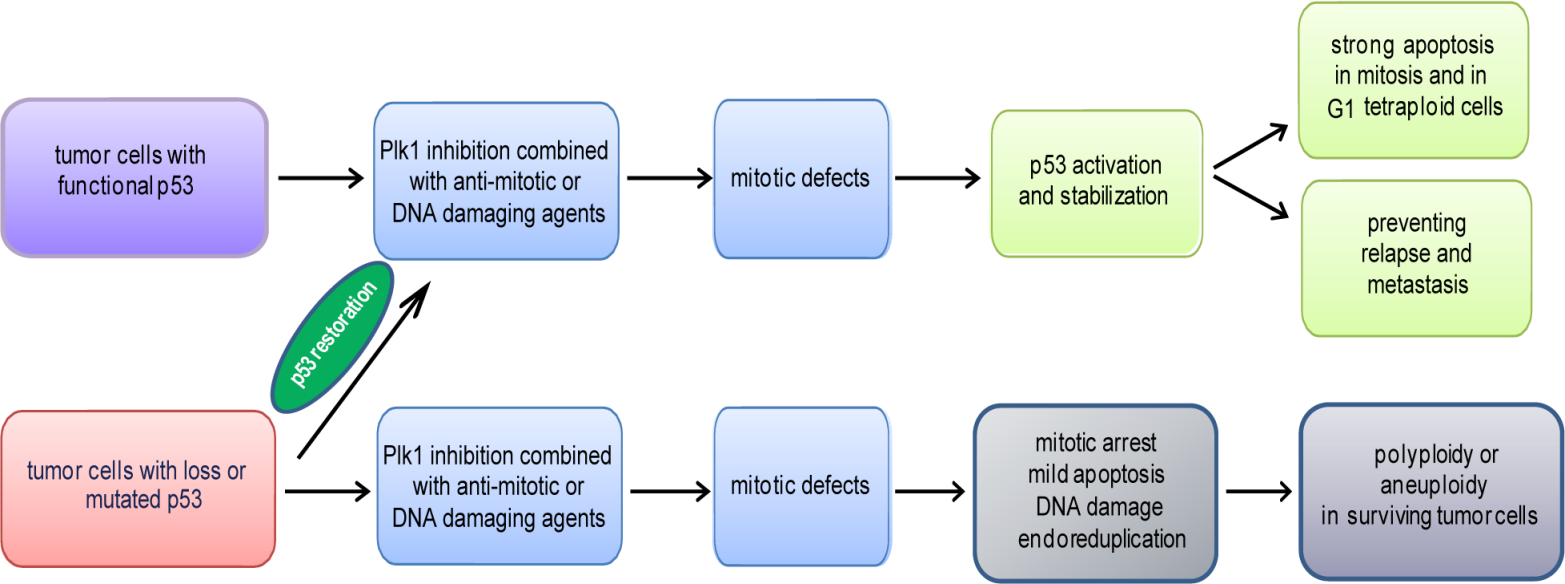
Schematic illustration of the impact of the p53 status on the efficacy of Plk1 inhibition Mono-therapy of Plk1 inhibition shows a moderate effect in clinical trials, suggestive of combined therapy with other agents, such as anti-mitotic or DNA damaging drugs. Tumor cells with functional p53 respond to Plk1 combined therapy with severe mitotic defects, activation of p53 followed by strong apoptosis in mitosis and in G1 tetraploidy. As p53 is involved in the regulation of the self-reprogramming of cancer stem cells and Plk1 inhibitors target the tumor initiating cells, it is conceivable to suggest that this strategy could empower cancer therapy by preventing relapse and metastasis. p53 restoration in tumor cells with loss or mutated p53 will reinforce the efficacy of Plk1 combined therapy. Otherwise, upon Plk1 combined therapy, tumor cells without functional p53 exhibit a modest apoptosis, DNA damage in mitosis, a longer mitosis linked to endoreduplication, which could make surviving tumor cells more malignant.

Plk1 and p53 intertwine and suppress each other in many signaling pathways. In the context of the association between the p53 status and Plk1 inhibition, we have addressed two issues: first, targeting Plk1 impacts actually all rapidly dividing cells irrespective of tumor cells or normal cells, which is in line with the crucial role of Plk1 in mitosis; second, tumor cells with functional p53 exhibit a stronger apoptosis than tumor cells with inactive p53. In addition, compared to the mono-therapy, combination of Plk1 inhibition with anti-mitotic or DNA damage agents induces more severe mitotic defects followed by apoptosis, and inhibits more strongly proliferation of cancer cells with functional p53. In this regard, restoration of p53 in tumor cells with loss or mutation of p53 will promote the cytotoxicity of combined Plk1 therapy, prevent genome instability induced by Plk1 inhibitors, and provide an effective strategy for combating relapse and metastasis of cancer (Fig. 3). A better understanding is required to maximize efficacy and minimize side-effects of combined Plk1 therapy in terms of administration schedules, such as succession and dosage, in each individual tumor.
